# NMR backbone assignment of the Cε4 domain of immunoglobulin E

**DOI:** 10.1007/s12104-020-09936-9

**Published:** 2020-02-27

**Authors:** Stefi V. Benjamin, Paul I. Creeke, Alistair J. Henry, James M. McDonnell

**Affiliations:** 1 Randall Centre for Cell & Molecular Biophysics, King’s College London, New Hunt’s House, Guy’s Campus, London, SE1 1UL UK; 2 UCB Pharma, 216 Bath Road, Slough, Berkshire SL1 3WE UK

**Keywords:** Immunoglobulin E, Cε4, Homodimer, Backbone assignment, NMR

## Abstract

Immunoglobulin E (IgE) plays a central role in allergic reactions. IgE is a dynamic molecule that is capable of undergoing large conformational changes. X-ray crystal structures of the Fc region of IgE in complex with various ligands have shown that IgE-Fc can exist in extended and various bent conformations. IgE-Fc consists of three domains: Cε2, Cε3 and Cε4. While the complete NMR backbone assignments of the Cε2 and Cε3 domains have been reported previously, the Cε4 domain has not been assigned. Here, we report the complete backbone assignment of the Cε4 homodimer. Cε4 can be used as a model system to study dynamics and allostery in IgE, as both molecules exist as homodimers and exhibit similar binding properties to a number of ligands.

## Biological context

Immunoglobulin E (IgE) is the central effector molecule of allergic reactions (Sutton and Gould 1993). IgE has two cellular receptors: FcεRI (Kinet 1999) and CD23 (Conrad 1990). An allergic reaction is initiated by allergen-mediated crosslinking of FcεRI-bound IgE, leading to activation of effector cells (mast cells, basophils, etc.) and the subsequent release of inflammatory mediators (Sutton and Gould 1993). The IgE molecule consists of two identical heavy chains and two identical light chains.

The ε heavy chains of IgE are comprised of four immunoglobulin (Ig) constant domains (Gould and Sutton 2008). Like other antibodies, the IgE structure can be divided into the Fab and Fc regions. The Fc region of the IgE molecule (IgE-Fc) interacts and binds to the IgE-specific receptors, FcεRI and CD23. IgE-Fc consists of three domains: Cε2, Cε3 and Cε4. The dimerisation of the ε heavy chain is mediated by a pair of intermolecular disulfide bonds between Cε2 domains and an extensive set of non-covalent interactions across the Cε4 dimer interface.

X-ray crystal structures of IgE-Fc have shown that the molecule exists in extended and various bent conformations (Drinkwater et al. 2014; Davies et al. 2017; Chen et al. 2018; Wan et al. 2002) when bound to different ligands. Kinetic analyses of IgE binding events by stopped-flow fluorescence and surface plasmon resonance confirm ligand-mediated conformational changes within the Fc region of IgE (Drinkwater et al. 2014).

Within IgE-Fc, solution state structural studies of the individual domains provide insights into the allosteric changes that are undergone by the molecule upon ligand binding. Although the backbone assignments of the Cε2 and Cε3 monomers have been completed using solution state NMR techniques (McDonnell et al. 2001; Borthakur et al. 2012), the backbone assignment of Cε4 has not been published. Here, we present the backbone NMR assignment for the non-covalent dimer of Cε4.

## Methods and experiments

The IgE Cε4 sequence was cloned into the pET15b-NTH vector. The resulting plasmid pET15b-NTH-Cε4 was transfected into BL21 (DE3) cells (NEB, UK). The recombinant strains were grown in M9 minimal media containing 0.07% ^15^NH_4_Cl and 0.2% ^13^C_6_-glucose (product code: 389374, Sigma-Aldrich) for ^15^N/^13^C-labeling of Cε4, or M9 media containing 100% D_2_O, and the same concentrations of isotope labelled nitrogen and carbon as above, for ^2^H/^15^N/^13^C-labeling of Cε4. Once an OD_600_ of 0.8 was reached, expression was induced using a final concentration of 1 mM IPTG. Following overnight incubation at 37 °C, the cells were harvested by centrifugation at 4000×*g* for 20 min. Cε4 was expressed in inclusion bodies, and extracted using a cell disruptor (Constant Systems Ltd.), following the protocol described by Taylor et al. (1992). A protease inhibitor cocktail was included during extraction (product code: S8830, Sigma-Aldrich) and Nonidet P40 substitute (code: 74385, Fluka, Biochemika) was used as the detergent.

The protein was refolded using the same protocol, concentrated using a cross-flow concentrator (Vivaflow 200, 3000 MWCO, PES membrane, Sartorius) and dialysed into 20 mM Na_2_HPO_4_ and 50 mM NaCl, pH 6.0. The Cε4 construct prepared here, and used for the NMR studies, contained an additional 20 residues comprising of a N-terminal hexa-His tag and a thrombin cleavage site.

In solution, Cε4 exists as a non-covalently bound homodimer, with a molecular weight of 29.2 kDa. There are 130 residues in this construct including the N-terminal hexa-His tag and thrombin cleavage site. The numbering of the Cε4 residues in this paper follows that used for the complete IgE molecule by Dorrington and Bennich (1978). Therefore, the first residue of Cε4 is G436 and the Cε4 sequence ends at residue K545. The residues that form the tag are labelled as − 20 to − 1, starting from the first residue in the tag to the last residue, respectively.

NMR spectra were recorded at 298 K on Bruker DRX700 and DRX800 spectrometers equipped with cryo-probes. The chemical shifts of ^1^HN, ^15^N, ^13^C_α_, ^13^C_β_ and ^13^CO cross peaks were assigned using a combination of HN(CO)CACB (Grzeseik and Bax 1992b), CBCA(CO)NH (Grzeseik and Bax 1992b), HNCACB (Grzeseik and Bax 1992a), HNCA (Kay et al. 1990), HN(CO)CA (Bax and Ikura 1991), HNCO (Kay et al. 1990) and HN(CA)CO experiments (Clubb et al. 1992). Several of these experiments were recorded using both ^2^H/^15^N/^13^C-labeled and ^1^H/^15^N/^13^C-labeled Cε4 samples. For some of the data, we found that experiments with the deuterated sample helped to resolve ambiguities in the Cε4 assignments.

## NMR assignments and data deposition for Cε4

**Fig. 1:**
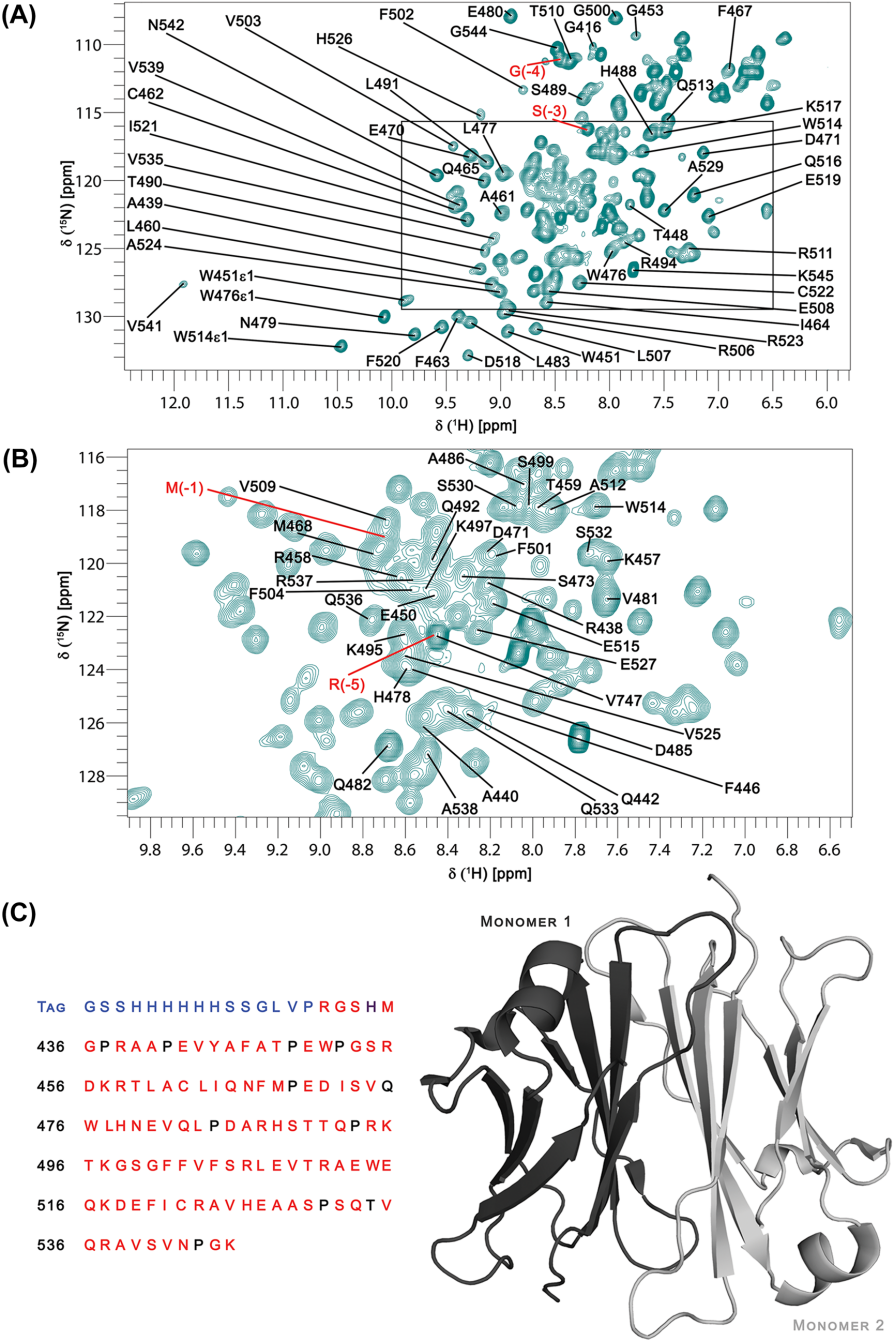
**a** The ^15^N-HSQC of Cε4 where the peaks are labelled with their residue assignment. The assignments for the peaks in the region labelled with a rectangle are shown in **b**. In both **a** and **b**, the assigned peaks are labelled with the residues in the tag labelled in red. (**c**, left panel) shows the sequence of Cε4 with the unassigned residues in the tag coloured blue, the assigned residues coloured red and the unassigned residues coloured black and (**c**, right panel) a cartoon representation of the structure of Cε4 (from PDB: 1O0V). One monomer is coloured dark grey and the other is coloured light grey. The ^15^N-HSQC of Cε4 was generated using CPPNmr Analysis and the cartoon illustration of Cε4 was generated using PyMOL. The figures were annotated using Adobe Photoshop (Adobe)

Backbone assignment of Cε4 was initially performed semi-automatically using CCPNmr Analysis (Vranken et al. 2005) then subsequently confirmed and completed manually. The cross peaks corresponding to the first 20 residues in the construct, forming the hexa-His tag and the thrombin cleavage site, were identified straightforwardly by clear differences in dynamics based on T_1_ and T_2_ relaxation studies and heteronuclear NOE experiments. Identification of these peaks was not part of the Cε4 backbone assignment process and the data are not reported here. Illustrated in Fig. 1c, the residues that are unassigned and correspond to the tag and cleavage site are coloured blue, the residues that have been assigned are coloured red and unassigned residues are coloured black.

98% of all possible amide H and amide N atoms (excluding the prolines), 99% of all possible Cα atoms, 94.5% of all possible Cβ atoms and 98.2% of all possible CO atoms were assigned. In total, ~ 96% of all possible backbone atoms (amide H, amide N, Cα, Cβ and CO), excluding the prolines, were assigned. The backbone assignment for Cε4 has been deposited with the BMRB database (BMRB entry 50091). Figure 1a and b show the assigned ^15^N, ^1^H-HSQC spectrum.

The amide proton of V541 resonates at an unusual downfield-shifted position compared with the other structured amides. This residue is located in a loop region near the C-terminal end of the protein. The crystal structure of IgE-Fc (PDB: 1O0V) shows that the amide proton of V541 is close to the aromatic rings of residues W478 and F520 (Wan et al. 2002). This likely leads to a significant shielding ring current effect for the amide proton of V541, resulting in this unusual chemical shift.

**Fig. 2:**
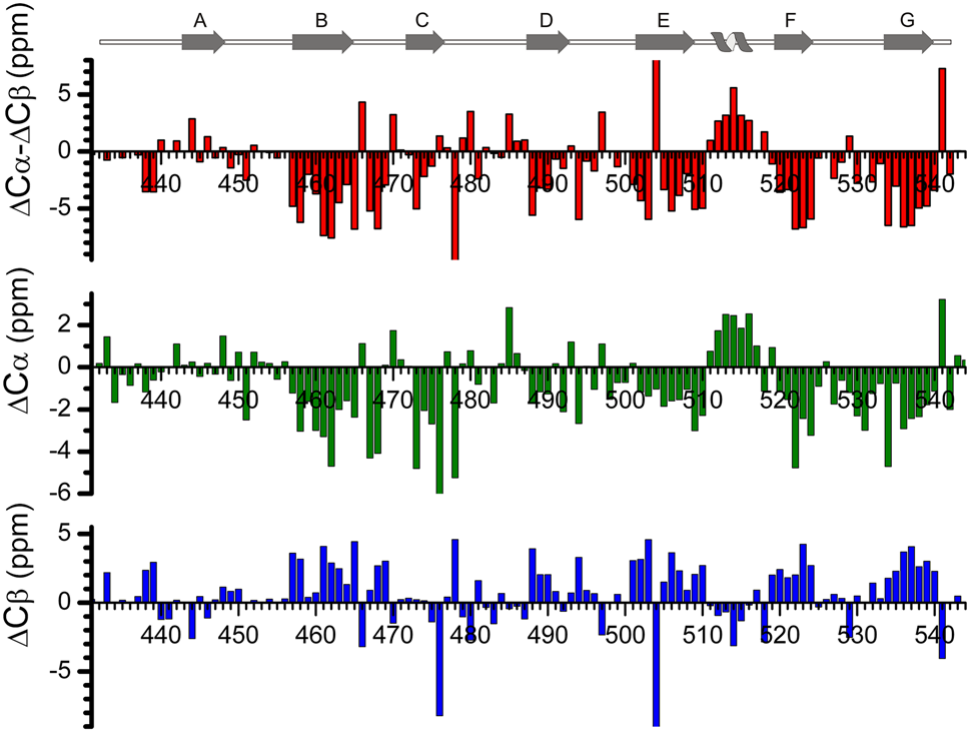
Figure showing the secondary chemical shifts for Cε4, based on the Cα and Cβ chemical shifts of Cε4. The figure also shows the secondary structure of Cε4 based on the IgE-Fc crystal structure (PDB: 1O0V)

Figure 2 shows a plot of the secondary ^13^C chemical shifts versus the Cε4 protein sequence (Wishart et al. 1992). This plot indicates unusual values for three residues: H478, F504 and V541. Firstly, both the Cα and Cβ values for H478 are unusual and this is because H478 is packed against the aromatic ring of F504, as observed in the crystal structure of IgE-Fc (PDB: 1O0V). The proximity of these atoms to the aromatic ring results in unusual chemical shift values. Secondly, the aromatic ring of F504 stacks above the aromatic ring of the F504 residue from the second monomer in the dimer interface. This proximity of the Cβ atom to the aromatic ring of the residue results in an unusual Cβ value. Finally, the chemical shift values for Cα and Cβ for V541 are unusual due to the close proximity of these atoms to the aromatic ring of W514.

A comparison of the secondary structure elements observed in the secondary chemical shift plot and that observed in the crystal structure shows that they are in good general agreement, with the exception of strand A. This is because strand A is an edge strand; an edge strand is defined as a beta strand that has inter-strand hydrogen bonds only on one side. Edge strands typically contain an unusual pattern of secondary chemical shifts that alternate between upfield and downfield shifted values (Hafsa et al. 2015), as observed for strand A (Fig. 2).

## Conclusion

The backbone assignment of Cε4 provides insight into the solution state structure of this domain. Since Cε4 was studied as a homodimer, it acts as a model system to study IgE-Fc, which also exists as a homodimer in solution. The backbone assignment data for Cε4 reported in this paper can be used to understand dynamics and allostery of the protein upon ligand binding.
